# Conversion of bone marrow mesenchymal stem cells into type II alveolar epithelial cells reduces pulmonary fibrosis by decreasing oxidative stress in rats

**DOI:** 10.3892/mmr.2014.2981

**Published:** 2014-11-19

**Authors:** KUN HUANG, XIAOWEN KANG, XINYAN WANG, SHIJIE WU, JINLING XIAO, ZHAOGUO LI, XIAOMEI WU, WEI ZHANG

**Affiliations:** 1 Department of Respiratory Medicine, The Second Affiliated Hospital, Harbin Medical University, Harbin, Heilongjiang 150081, P.R. China; 2 Department of Respiratory Medicine, Daqing Oilfield General Hospital, Daqing, Heilongjiang 163316, P.R. China; 3 Department of Respiratory Medicine, The First Affiliated Hospital, Harbin Medical University, Harbin, Heilongjiang 150001, P.R. China

**Keywords:** pulmonary fibrosis, bone marrow mesenchymal stem cells, type II alveolar epithelial cells, oxidative stress

## Abstract

Pulmonary fibrosis is an irreversible chronic progressive fibroproliferative lung disease, which usually has a poor prognosis. Previous studies have confirmed that the transplantation of bone marrow mesenchymal stem cells (MSCs) significantly reduces lung damage in a number of animal models. However, the underlying mechanism involved in this process remains to be elucidated. In the present study, a bleomycin (BLM)-induced female Wister rat model of fibrosis was established. At 0 or 7 days following BLM administration, rats were injected into the tail vein with 5-bromo-2-deoxyuridine-labeled MSCs extracted from male Wistar rats. The lung tissue of the rats injected with MSCs expressed the sex-determining region Y gene. The level surfactant protein C (SP-C), a marker for type II alveolar epithelial cells (AEC II), was higher in the group injected with MSCs at day 0 than that in the group injected at day 7. Furthermore, SP-C mRNA, but not aquaporin 5 mRNA, a marker for type I alveolar epithelial cells, was expressed in fresh bone marrow aspirates and the fifth generation of cultured MSCs. In addition, superoxide dismutase activity and total antioxidative capability, specific indicators of oxidative stress, were significantly increased in the lung tissue of the MSC-transplanted rats (P<0.05). In conclusion, to alleviate pulmonary fibrosis, exogenous MSCs may be transplanted into damaged lung tissue where they differentiate into AEC II and exert their effect, at least in part, through blocking oxidative stress.

## Introduction

Pulmonary fibrosis is a progressive lung disease characterized by critical injury of functional alveolar epithelial cells and the excessive production of extracellular matrix proteins (1–3). A number of novel therapeutic approaches are emerging, following significant progress in the understanding of the molecular events associated with fibrosis, which involve cytokines (4–6), growth factors (7) and biochemical signaling pathways (6,8). However, there are currently no effective treatments to reverse or delay the course of this disease (9,10). The pathogenesis of pulmonary fibrosis remains elusive; however, oxidative stress, hyperplasia, denudation and apoptosis of type II alveolar epithelial cells (AEC II) may be important early features in this condition (11,12).

There is increasing evidence that oxidative stress is involved in the pathogenesis of pulmonary fibrosis. The development of bleomycin (BLM)-induced pulmonary fibrosis is dose-dependent and may occur due to an increase in the concentration of reactive oxygen species (ROS) (13). Markers of oxidative stress have been identified, demonstrating that aberrant antioxidant activity may exacerbate pulmonary fibrosis in patients and animal models (12,14). AEC II cells, which are known to be vulnerable to oxidative stress, produce and secrete the surfactant proteins (SPs), SP-A, SP-B, SP-C and SP-D (markers of alveolar epithelial cell function) in order to maintain morphological organization, biophysical functions, biochemical composition and immune function in lung tissue (15).

Bone marrow mesenchymal stem cells (MSCs) are adult stem cells that are present in the stroma of bone marrow and certain other organs (16,17). Previous studies have shown that MSCs have potential as regenerative therapeutics due to their strong proliferative capacity and differentiation potential (18,19). With the capacity for mesoderm-like cell differentiation into, for example, adipocytes, osteoblasts and chondrocytes, MSCs are able to repair damage in a number of tissues (20). Stem cell therapy may prove to be effective in treating lung disease. However, the mechanisms underlying this effect have not yet been fully elucidated (21–23).

The present study hypothesized that systemically transplanted exogenous MSCs migrate into damaged lung tissue and develop functional characteristics of alveolar cells, thus slowing the progression of pulmonary fibrosis. In addition, it was postulated that this effect is associated with changes in oxidative stress. To test these hypotheses, the effect of systemic MSC transplantation on the lung tissue of a rat model of BLM-induced pulmonary fibrosis was investigated at a number of time-points (24). Reverse transcription polymerase chain reaction (RT-PCR) and immunofluorescence staining were used to detect male rat Y chromosome-specific sequences in the recipient female rat lung tissue (25) and to assess the differentiation of MSCs into alveolar cells. Experiments to assess superoxide dismutase (SOD) activity and total antioxidative capability (T-AOC) were also conducted.

## Materials and methods

### Animals

Pathogen-free Wistar rats were obtained from the Animal Testing Center, The Second Affiliated Hospital of Harbin Medical University, (Harbin, China). Their use was approved by the Animal Care and Use Committee of Harbin Medical University. All animals were kept in accordance with the National Institutes of Health Guidelines on the Use of Laboratory Animals. Rats were fed with a conventional laboratory diet, with ad libitum access to tap water in an air-conditioned room at ~25°C.

### Isolation, identification and labelling of MSCs

MSCs from a four-week-old clean-grade male Wistar rat were plated into tissue culture flasks. Adherent cells were allowed to grow to ~75% confluency, trypsinized and reseeded at a density of 10^5^ cells/cm^2^ (26). This procedure was performed for four passages. When the 5th generation of cells reached 50–60% confluency, 5-bromo-2-deoxyuridine (BrdU; Sigma-Aldrich, St. Louis, MO, USA) was added to obtain a final concentration of 10 μmol/l. These cells were further incubated for 48 h (27). Following digestion and centrifugation at 1,000 × g for 5 min, cells were washed in a flow cytometry buffer (Becton Dickinson, San Jose, CA, USA) and incubated for 30 min with fluorescein-5-isothiocyanate (FITC)-conjugated anti-rat monoclonal antibodies, including anti-CD44, anti-CD45 and anti-CD90 (1:100, BioLegend, Inc., San Diego, CA, USA). Washed cells were pelleted and resuspended, and fixed in 1% paraformaldehyde for 15 min. Non-specific fluorescence was determined using equal aliquots of the cell preparation incubated with anti-mouse monoclonal antibodies (1:4,000, BioLegend, Inc.). Data were acquired and analyzed on FACSCalibur with CellQuest software (Becton Dickinson) (28). Fluorescein isothiocyanate (FITC)-conjugated anti-rat CD44, CD45 and CD90 antibodies were obtained from BioLegend, Inc. (San Diego, CA, USA). A cell suspension with a live cell density of 2.5×10^6^ cells/ml was prepared with serum-free Dulbecco’s modified Eagle’s medium (DMEM) for transplantation (29). Flasks, trypsin and DMEM were obtained from Gibco Life Technologies (Carlsbad, CA, USA). Paraformaldehyde, chloral hydrate, NaCl, H_2_O_2_, PBS, phenol, chloroform and agarose were all obtained from Sigma-Aldrich (St. Louis, MO, USA).

### Establishment of a rat pulmonary fibrosis model and delivery of MSC transplantation

A total of 48 clean-grade female Wistar rats (weight, 200–240 g) were randomly divided into four groups: BLM control group; immediate MSC injection group (BLM with administration of MSCs at day 0); 7-day MSC injection group (BLM with administration of MSCs at day 7); and negative control group (12). Rats in the BLM group were given a slow intratracheal injection of BLM (Nippon Kayaku Co., Ltd., Tokyo, Japan) at a dose of 5.0 mg/kg following intraperitoneal anesthesia with 10% chloral hydrate (0.03 ml/kg). Following the injection, rats were held upright and rotated in order to distribute the drug as evenly as possible. In the immediate MSC injection group and the 7-day MSC injection group, rats were injected with 1 ml BrdU-labeled MSCs from a male rat (containing 2.5×10^6^ cells) into the tail vein, at days 0 and 7 following BLM administration, respectively. The negative control rats received an intratracheal injection of 0.9% NaCl. On days 7, 14 and 28, four rats from each group were sacrificed with 10% chloral hydrate (0.3 ml/100 g) by intraperitoneal injection.

### Histopathological analysis

The lung tissue was fixed in 4% neutral-buffered formaldehyde for paraffin embedding, cut into 4-μm sections. Paraffin sections were de-waxed twice with dimethyl benzene at 37°C for 15 min, then soaked in 95, 70 and 30% ethanol, and distilled water for 2 min each, and washed twice in water (37°C) for 45 sec. Each section was stained with hematoxylin and eosin (H&E) and Masson trichrome (Jiancheng Co., Ltd., Nanjing, China). The remaining reagents were obtained from Beyotime Institute of Biotechnology (Haimen, China). Standard scoring using the Szapiel score was implemented for the semiquantitative assessment of the severity of alveolitis and pulmonary fibrosis as described previously (30). All histological specimens were coded and interpreted in a blinded fashion. Three sections were selected, and ten visual fields from each section were observed. Images were evaluated by two assessors, and were captured by an Optical Microscope (Olympus CX41, Olympus, Tokyo, Japan).

### Determination of hydroxyproline content

The collagen content in the lung tissue was measured using the hydroxyproline (Hyp) assay as described previously (31). Hyp content was measured using a Hyp detection kit (Jiancheng Biotechnology Institute, Nanjing, China) according to the manufacturer’s instructions.

### Immunofluorescence staining

Following sacrificing the rats, part of the right lower lobes and part of the left lungs were removed, embedded in polyvinyl alcohol and stored in liquid nitrogen. Serial sections of ~6 μm were prepared using a cryostat. The frozen sections were treated with 3% H_2_O_2_ in order to block endogenous peroxidase. Following DNA denaturation, 0.1% trypsin pre-treatment was added. Normal goat serum (1:20; Sigma-Aldrich) was used for blocking for 20 min. Subsequently, mouse monoclonal anti-BrdU primary antibody (BU33, 1:100, Sigma-Aldrich) with or without rabbit anti-SP-C polyclonal antibody (FL-197, 1:200, Santa Cruz Biotechnology, Inc., Dallas, TX, USA) was added, tissue sections were incubated overnight in a 4°C humidity chamber and washed once with phosphate-buffered saline (PBS). Non-specific serum, in place of the primary antibody, was used as the negative control. An FITC-labeled goat anti-mouse secondary antibody (1:100, Santa Cruz Biotechnology, Inc.) and a tetramethylrhodamine-labeled goat anti-rabbit immunoglobulin G secondary antibody (1:100, Santa Cruz Biotechnology, Inc.) were added. Tissue sections were then incubated at 37°C in the dark for 1 h and washed once with PBS. DAPI (Beyotime Institute of Biotechnology), 1:1,000 was added in order to restain the nucleus and slides were then mounted. The staining results were observed and radiographed using fluorescence microscopy (Nikon MF30 LED, Nikon Corporation, Tokyo, Japan). Four to six fields of view for each specimen were randomly selected and observed under the microscope. The procedures for SP-C immunofluorescence staining of fresh bone marrow aspirates and cultured MSCs of the fifth generation were identical to those described for assessment of the lung tissue, with the exception of the use of 4% paraformaldehyde as the fixative solution.

### PCR detection of sex-determining region Y (SRY) gene

Data from a previous study (32) was used to identify and synthesize the rat SRY gene product. The following primers were used: Forward, 5′-TTTAGTGTTCAGCCTACAGCC-3′ and reverse, 5′-GCACTTTAACCCTCGATGAGG-3′ for SRY, and forward, 5′-CACGATGGAGGGGCCGGACTCATC-3′ and reverse, 5′-TAAAGACCTCTATGCCAACACAGT-3′ for β-actin. Primers were synthesized by Chaoshi Biotech Co. (Shanghai, China).” The lengths of the amplified fragments for SRY and β-actin were 322 and 240 bp, respectively. DNA from the lung tissue of female rats was extracted using phenol and chloroform. PCR conditions were as follows: Initial denaturation at 94°C for 5 min, denaturation at 94°C for 30 sec, annealing at 52°C for 30 sec, extension at 72°C for 30 sec for 30 cycles and full extension at 72°C for 5 min. Gel electrophoresis in 2% agarose was conducted in order to analyze the PCR products. An image scanner was used to radiograph and analyze the average grayscale of target bands (TannonGIS gel imaging and analysis system; Biotanon Biotech Co., Shanghai, China). The results were compared with those of the female negative control and male positive control groups.

### RT-PCR analysis of SP-C and aquaporin 5 (AQP-5) mRNA expression

RNA from 100 mg lung tissue, (1–2)×10^7^ fresh bone marrow aspirates and 5×10^6^ fifth generation MSCs was extracted using a TRIzol^®^ total RNA extraction kit (Invitrogen Life Technologies, Carlsbad, CA, USA). Primers were synthesized by Shanghai Chaoshi Biotech Co. (Shanghai, China). The following primers were used: Forward, 5′-GTCCTTGTCGTCGTGGTGAT-3′ and reverse, 5′-AGGTAGCGATGGTGTCTGTGT-3′ for SP-C, and forward, 5′-AACCCAGCCCGATCTTTC-3′ and reverse, 5′-GGAAGAGCAGGTAGAAATAGAGG-3′ for AQP-5. The lengths of the amplified fragments for SP-C and AQP-5 were 154 bp and 424 bp, respectively. RT-PCR was conducted using a one-step reaction system kit (Takara Biotechnology Co., Ltd., Dalian, China), which consisted of complementary DNA (cDNA) synthesis and pre-denaturation using the following conditions: Reverse-transcription at 42°C for 30 min and 99°C for 5 min for one cycle, PCR amplification with denaturation at 94°C for 30 sec, annealing at 52°C for 30 sec, extension at 72°C for 30 sec for 30 cycles and final extension at 72°C for 5 min. Gel electrophoresis and semi-quantitative mRNA detection were conducted using 2% agarose gel electrophoresis of 10 μl of each of the amplified SP-C, AQP-5 and β-actin products, followed by radiography using an image scanner (WD-9413A, Liuyi Instrument Factory, Beijing, China).

### Measurement of SOD activity and T-AOC

SOD and T-AOC kits were purchased from Nanjing Jiancheng Bioengineering Institute (Ninjing, China). BLM-injected rats were treated with immediate injection of MSCs or with no treatment and lung tissues were collected and homogenized. The lung homogenates were centrifuged for 30 min at 3,000 × g at 4°C. The content of protein in the supernatant was determined using a Bradford protein assay kit (Beyotime Institute of Biotechnology). The procedures used to quantify SOD activity and T-AOC were conducted according to the manufacturer’s instructions on a plate reader (Tecan Infinite 200, Eastwin Life Science, Beijing, China). The absorbance change at 520 and 550 nm was monitored (33,34). Data are expressed as the mean absorbance normalized to the percentage of the vehicle control.

### Statistical analysis

Experimental data are expressed as the mean ± standard error of the mean. SPSS version 17.0 (SPSS, Inc., Chicago, IL, USA) was used to calculate analysis of variance in order to compare the data between groups. P<0.05 was considered to indicate a statistically significant difference.

## Results

### Identification and labelling of MSCs

In the present study, the labeling rate of MSCs accounted 98% after MSCs were incubated with BrdU (10 μmol/) for 48 h. Furthermore, the analysis of the cell surface phenotype indicated that the MSC population was positive for CD44 and CD90 but negative for CD45, which was consistent with the International Society for Cellular Therapy position statement for the minimum criteria for an MSC (28).

### MSCs reduce alveolitis and pulmonary fibrosis in the injured lung

**Figure 1:**
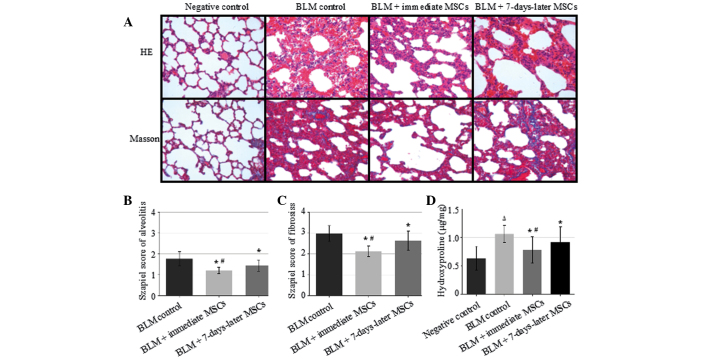
Effect of transplantation of exogenous MSCs on BLM-induced alveolitis and pulmonary fibrosis. (A) Representative histological lung sections from each group. Tissues were stained with HE and Masson trichrome (for the assessment of collagen content). Blue staining represents collagen deposition (Masson trichrome). Alveolitis was assessed by evaluation of inflammatory cell infiltration and pulmonary fibrosis was assessed by evaluation of interstitial collagen deposition on day 28 following BLM administration. Original magnification, ×200; (B) Szapiel scores for alveolitis on day 28 following BLM in each group. (C) Szapiel scores for pulmonary fibrosis on day 28 following BLM administration in each group. (D) Collagen content was measured on day 28 following BLM administration using a hydroxyproline assay. Data are presented as the mean ± standard error of the mean for four rats per group. ^Δ^P<0.01 compared with the negative control group, ^*^P<0.05 compared with the BLM group and ^#^P<0.05 compared with the 7-day MSCs injection group. MSCs, mesenchymal stem cells; BLM, bleomycin; HE, hematoxylin and eosin.

Rat lung sections from each group on day 28 are shown in Fig. 1A. There was no obvious lesion in the lungs of rats from the negative control group. In the BLM control group, H&E staining showed that the pulmonary alveolus cavity was markedly decreased in size, the alveolar wall was thickened, and the mesenchyme was expanded, with increased numbers of fibroblasts and macrophages compared with the negative control group. Masson trichrome staining showed that collagen was increased in the lung interstitium, as well as around blood vessels and bronchi, when compared with the negative control group. In the immediate MSCs group, the majority of alveoli were intact. The thickness of the alveolar wall, the number of fibroblasts and the quantity of collagen in the lung interstitium were significantly reduced compared with the those in the BLM control group. The Szapiel score for alveolitis and pulmonary fibrosis on day 28 following BLM administration were significantly reduced by transplantation of MSCs at day 0 and day 7, compared with those in the BLM control group (P<0.05). Administration of MSCs at day 0 further decreased the Szapiel score of alveolitis and pulmonary fibrosis compared with treatment at day 7 (P<0.05; Fig. 1B and C). Furthermore, according to the Hyp content on day 28, there was a significant difference in the lung collagen content between the immediate MSCs group and the BLM control group (P<0.05; Fig. 1D).

### Transplantation of the exogenous MSCs

**Figure 2:**
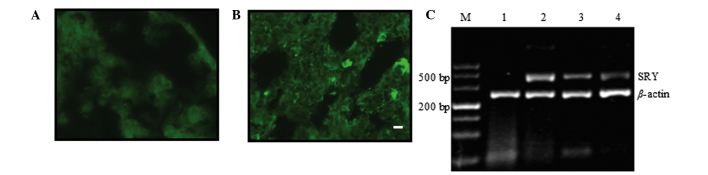
MSCs from male rats were transplanted into BLM-induced female rat models of pulmonary fibrosis. (A and B) Donor 5-bromo-2-deoxyuridine-labelled MSCs (green) were observed in recipient lung tissue 28 days following injection of MSCs. (A) Negative control group and (B) immediate MSCs injection group. Scale bars, 10 μm; original magnification, ×400. (C) Polymerase chain reaction analysis of the SRY gene in rat lung tissue. Lane M, DL500 DNA marker; lane 1, control female rats; lane 2, control male rats; lane 3, immediate MSCs injection group; and lane 4, 7-day MSCs injection group. MSCs, mesenchymal stem cells; BLM, bleomycin; SRY, sex-determining region Y gene.

To detect whether BrdU-labelled exogenous MSCs migrated to damaged lung tissue, stand-alone BrdU staining was used. BrdU-labelled MSCs were identified in the frozen sections of lung tissue from rats sacrificed on days 7, 14 and 28. These cells showed green fluorescence. MSCs were predominantly distributed as single cells. Clusters of two to three cells were also present. The shapes of the MSCs were predominantly oval or cubic, and a number were spindle-shaped at one end. The nuclei were round or oval in shape and were relatively large. Morphologically, the MSC distribution was consistent with the usual location and size of AEC II. At the earlier time-points, MSCs were primarily distributed around the interstitial lung and pulmonary blood vessels. However, at day 28, MSCs were aggregated and were principally distributed in the alveolar walls, with a number of MSCs distributed in the collapsed alveolar spaces, in which the interstitial changes were greatest. In the 7-day MSCs injection group, a small number of BrdU-positive MSCs were observed. However, compared with the immediate MSCs injection group, the number was significantly smaller. No BrdU-positive cells were found in the negative control group (Fig. 2A and B).

To further verify that the transplanted MSCs had migrated into the lung tissue, PCR was used to detect the presence of the male SRY gene on the Y chromosome in lung tissue in which positive stand-alone BrdU staining was observed. The length of the SRY gene fragment used for detection was 322 bp. The length of the β-actin fragment, used as an internal control, was 240 bp. Following PCR amplification of the DNA that was extracted from rat lung tissue, electrophoresis demonstrated only a single 240 bp internal control band in the female negative control rats, but an additional 322 bp gene band in the male positive control rats. The 322 bp bands were also detected in the immediate MSCs injection group and in the 7-day MSCs injection group, which each exhibited identical electrophoresis patterns to those of the male positive control male rats. However, the levels of SRY gene expression, as measured by the average greyscale of the target bands, was significantly lower in the 7-day MSC injection group (0.04) than in the immediate MSC injection group (0.23; P<0.05; Fig. 2C).

### Dual immunofluorescence staining of lung tissue

**Figure 3:**
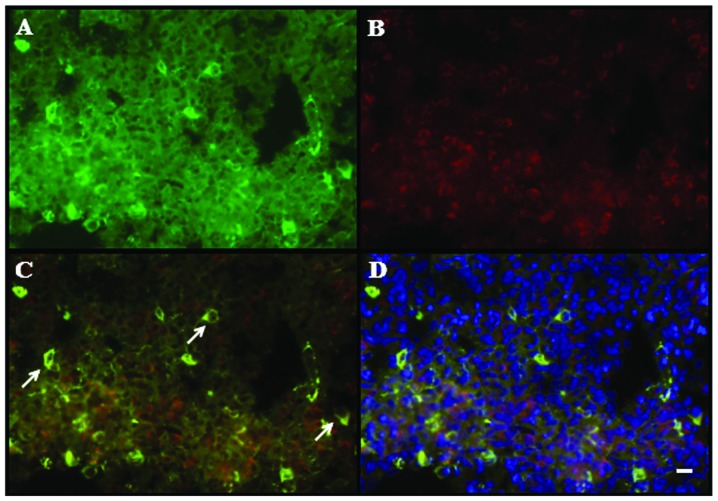
Double labeling for BrdU and SP-C expression in recipient lungs from immediate MSCs injection group. The immunofluorescence of frozen lung sections was examined using a fluorescence microscope. (A) Localization of BrdU (green), (B) localization of SP-C (red), (C) merge of (A) and (B) (yellow) and (D) nuclear staining (DAPI, blue). All donor-derived green cells are also positive for SP-C staining (arrows), identifying them as AEC II. Scale bars, 10 μm; original magnification, ×400. BrdU, 5-bromo-2-deoxyuridine; SP-C, surfactant protein-C; AEC II, type II alveolar epithelial cells.

To determine whether MSCs that had migrated into the lung tissue differentiated into AEC II and exhibited AEC II function, lung tissue was treated with dual immunofluorescence staining for SP-C and BrdU. As shown in Fig. 3, green fluorescence staining indicated BrdU-positive cells, red fluorescence in the cytoplasm indicated SP-C-positive cells and yellow fluorescence indicated BrdU- and SP-C-positive cells. Dual BrdU- and SP-C-positive cells were observed in frozen sections of lung tissue from the immediate MSC injection group on days 7, 14 and 28. The dual positive cells were predominantly distributed in the alveolar walls and a number were observed in the collapsed alveolar spaces, in which the interstitial changes were greatest. In addition, all the BrdU-positive cells in the lung tissue that were morphologically similar to AEC II, but different from AEC I and fibroblast cells, were able to differentiate into AEC II. A small number of dual positive cells were also be found in the rat lung tissue from the 7-day MSC injection group.

### SP-C and AQP-5 mRNA expression in different tissues and cells

**Figure 4:**
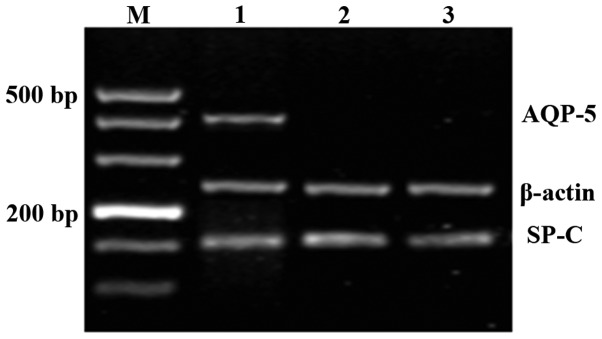
Reverse transcription-polymerase chain reaction analysis of the alveolar epithelial markers SP-C and AQP-5 from different tissues and cells. Lane M, DL500 DNA marker; lane 1, normal lung tissue; lane 2, fresh bone marrow aspirates; and lane 3, cultured mesenchymal stem cells of the fifth generation. SP-C, surfactant protein-C; AQP-5, aquaporin 5.

It was shown that exogenous MSCs differentiated into AEC II in the lung tissue of recipients. In order to investigate at what point this differentiation occurred, the mRNA levels of SP-C, a specific marker for AEC II, and AQP-5, a specific marker for AEC I, were examined. As shown in Fig. 4, mRNA from each marker was expressed in normal lung tissue. However, in fresh bone marrow, aspirates and the fifth generation of cultured MSCs, only the 154 bp SP-C mRNA was expressed, as detected by RT-PCR. In order to further validate the SP-C expression in bone marrow stem cells, SP-C immunofluorescence staining of fresh bone marrow aspirates and the fifth generation of cultured MSCs was performed, and no positive cells were observed in these samples.

### Oxidative stress detection

**Figure 5:**
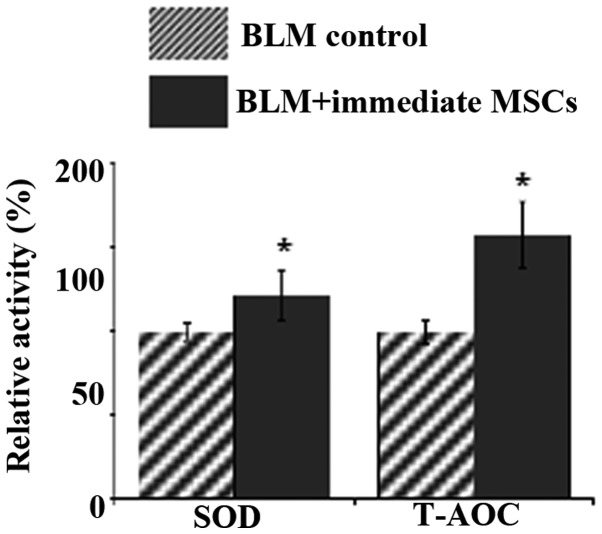
Oxidative stress in lung tissue of BLM-induced rat model of pulmonary fibrosis. Pulmonary SOD activity and T-AOC were detected by a colorimetric method in rats treated with or without immediate injection of MSCs. Data are presented as the mean ± standard error of the mean of three independent experiments. ^*^P<0.05, compared with the BLM control group. BLM, bleomycin; SOD, superoxide dismutase; T-AOC, total antioxidative capability; MSCs, mesenchymal stem cells.

It was hypothesized that the alleviating effect of pulmonary fibrosis following MSC injection may be partly due to the alleviation of the oxidative damage by AEC II, which MSCs differentiate into. SOD is an important component of the antioxidant enzymatic defense system and converts the superoxide radical to H_2_O_2_. T-AOC reflects the overall cellular endogenous antioxidative capability, including enzymatic as well as non-enzymatic antioxidants (35). SOD activity and T-AOC in the lung tissue from the immediate MSC injection group were significantly increased compared to those in the BLM control group (P<0.01, Fig. 5). Thus, AEC II generation as a result of injection of MSCs, significantly attenuated the oxidative stress in pulmonary fibrosis.

## Discussion

In the present study, bone marrow-derived MSCs were shown to differentiate into AEC II when transplanted into rats following BLM administration. In addition, their behavior was altered by the microenvironment of injury.

BLM is one of the most extensively studied and reproducible agents used to induce lung fibrosis in rats. When BLM is given into the airway, it causes lung epithelial injury, followed by an inflammatory response, which is followed by the development of lung fibrosis that eventually resolves (36). Purified bone marrow cells that proliferated *in vitro* exhibited morphological characteristics of MSCs, as indicated by a CD45^−^CD44^+^CD90^+^ cell surface phenotype. In the present study, when transplanted into Wistar rats with BLM-induced lung injury, MSCs were shown to promote the repair process following lung injury and fibrosis. The lung injury Szapiel score for inflammation and fibrosis on day 28 following BLM administration was significantly reduced by the transplantation of MSCs. Moreover, according to the Hyp content on day 28, there was a significant difference in the collagen content of the lungs of rats treated with MSCs compared with that in the BLM control group.

A characteristic function of mature AEC II is the synthesis and secretion of pulmonary SPs, including SP-A, SP-B, SP-C and SP-D. Notably, SP-C is exclusively expressed in AEC II and therefore may be used for the identification of these cells (37,38). AQP-5 is a specific marker for AEC I, although in the present study, dual AQP-5 and BrdU immunofluorescence staining was not performed as it was possible to differentiate AEC I from AEC II (39). AEC II have been postulated to act as stem cells in lung tissue (40), giving rise to AEC I in response to injury, and are known targets of apoptotic signals induced by lung lesions (39,41,42). By replacing AEC II, MSCs may limit the effects of lung injury, and their differentiation into AEC II may partially restore the stem cell pool.

The results of the present study demonstrated that when MSCs were administered immediately following the establishment of the BLM-induced animal model of fibrosis, exogenous MSCs were detected in lung tissues from rats killed on days 7, 14 and 28. Furthermore, the number of MSCs in the lung tissue increased over time. The distinct cellular localization, morphology and molecular features of the engrafted cells indicated that they expressed the type II pneumocyte phenotype, and that these cells had the SP-C secreting function characteristic of AEC II. However, when MSCs were administered at 7 days following the establishment of the animal model, only a small number of dual positive cells were detected in the lung tissue from rats killed on day 28. The PCR results confirmed that male MSCs migrated into the lung tissue of female recipients in varying quantities in the immediate and 7-day MSC groups.

Under normal conditions, MSCs exist in G_0_ or G_1_ phase of the cell cycle and are thus in a relatively static state. However, upon induction by certain factors, MSCs may be stimulated to differentiate and migrate to damaged tissues. Ortiz *et al* (22) showed that BLM-induced lung injury promoted the migration of transplanted MSCs into the lungs as well as the differentiation of MSCs into AEC II, resulting in repair of lung damage and a reduction in pulmonary fibrosis. However, a study by Kotton *et al* (21) produced different results. Bone marrow stem cells that had attached to petri dishes were cultured for one week and then transplanted into rats with BLM-induced lung injury. These cells were observed to migrate to the lungs and differentiate into AEC I. The morphological characteristics and molecular biological phenotypes of endogenous AEC I were also detected in fresh bone marrow aspirates and bone marrow cells that had been cultured for one week. Furthermore, cultured bone marrow-derived cells exhibited features of lung cell differentiation prior to engraftment. Therefore, results regarding the conditions of MSC migration and differentiation may markedly vary by experimental design. The present study showed that the morphological characteristics and the molecular biological phenotypes of endogenous AEC II were detectable in all BrdU-labelled MSCs following migration into recipient lung tissue. In fresh bone marrow aspirates and fifth generation of cultured MSCs, RT-PCR detected mRNA expression of the AEC II-specific marker SP-C, but not of the AEC I specific marker AQP-5. SP-C immunofluorescence staining did not detect SP-C-positive cells. The experimental design was considerably different from that of Ortiz *et al* (22), but nevertheless yielded identical results. By contrast, the experimental protocol was similar to that used by Kotton *et al* but yielded contradictory results. There are a number of possible reasons for these discrepancies. Kotton *et al* used bone marrow cells for transplantation. These had not been subcultured and purified, and were therefore not MSCs. It is currently understood that bone marrow cells contain three cell groups: Hematopoietic stem cells, MSCs and endothelial precursor cells (43). The morphological characteristics and molecular biological phenotypes of AEC I that were detected by Kotton *et al* were likely to have been produced by the endothelial precursor cells. In addition, AEC II are functionally equivalent to the stem cells of the lungs, in that they can reproduce to produce AEC II progeny or rapidly differentiate into AEC I. The AEC I detected by Kotton *et al* are likely to originate from differentiated AEC II.

Through differentiation into AEC II, MSCs may partially restore the lung stem cell pool. The proliferation of lung stem cells may also explain how the low number of MSCs that migrated to the lungs had such a marked effect on the health status of rats with BLM-induced lung damage. The results of the present study suggested that exogenous MSCs migrated into injured lungs and differentiated into AEC II. This differentiation potential was present prior to transplantation, but the cells required a local microenvironment in order to complete differentiation and to develop the morphological characteristics and function of AEC II. The MSC transplantation rate was higher when MSCs were injected in the early stages of lung injury. This increased efficacy may be associated with lung injury, and the chemokines produced by this injury, which in turn promote MSC migration into the lungs and the subsequent directional differentiation (44). MSC differentiation is key to the therapeutic role of these cells in pulmonary fibrosis.

Oxidative stress has been implicated as a possible molecular mechanism underlying fibrosis in a variety of organs, including the lung. Accumulating evidence also indicates that BLM induces lung injury as a result of its ability to generate ROS, including superoxide and hydroxy radicals (45). Accordingly, in the present study, the levels of markers of oxidative stress were investigated. It was shown that the antioxidant enzyme activities of SOD and T-AOC were significantly decreased in the immediate MSC injection group compared with those in the BLM control group. These results supported the hypothesis that the AEC II generation following injection of MSCs may responsible for the alleviation of pulmonary fibrosis, at least in part due blocking oxidative damage.

In the present study, MSCs were shown to differentiate into AEC II, suggesting that differentiation may be the primary mechanism through which MSCs exert a therapeutic effect. MSCs possess this differentiation potential at the bone marrow stage, but are only able to differentiate within a microenvironment of injury. The stem cells repair damage caused by BLM, at least in part, by blocking oxidative stress. Further studies are required to investigate alternative and additional mechanisms. In conclusion, MSC migration and differentiation may provide an effective cell therapy for pulmonary fibrosis.
